# Quantitative Estimation of Synergistic Toxicity of Cu and Zn on Growth of *Arabidopsis thaliana* by Isobolographic Method

**DOI:** 10.3390/toxics10040195

**Published:** 2022-04-16

**Authors:** Bumhan Bae, Hyesun Park, Sua Kang

**Affiliations:** Department of Civil & Environmental Engineering, Gachon University, Seongnam 13120, Korea; hsun1090@naver.com (H.P.); kangsua2630@naver.com (S.K.)

**Keywords:** *Arabidopsis thaliana*, combination index, EC50, heavy metal, photosynthesis pigments, total root length

## Abstract

Heavy metal is one of the most frequent soil contaminants and contaminated soils generally include numerous metals. Although exposure to multiple metals may increase the toxicity to humans and ecosystems, only additive effects are considered in the risk assessment. In this study, the synergistic effect of heavy metals (Cu and Zn) on a model plant, *Arabidopsis thaliana*, was quantified by the isobolographic method. The plant was cultured via the growth assay method on a plant agar containing individual heavy metals or combinations of Cu + Zn in a growth chamber. The concentration of Cu varied by eight levels from 0 to 200 μM and the concentration of Zn also varied by eight levels from 0 to 400 μM. In the combination of metals, each of the three levels of Cu (25–75 μM) and Zn (20–100 μM) were applied. After 8 days, plants were harvested for root/shoot weight and measured for leaf chlorophyll and carotenoid content. The primary and secondary root elongation of *A. thaliana* was estimated using image analysis to calculate total root length. The EC50 values of Cu and Zn on *A. thaliana*, based on the total root length, were 40.0 and 76.4 μM, respectively. When two heavy metals were administered in combination, the EC values decreased less than those of the individual metals. The average value of the combination index was 0.6, proving the synergistic toxic effect on the root growth of *A. Thaliana*. As a result, the isobolograhic method is a useful tool for estimating the quantitative toxic effect of chemicals on plants.

## 1. Introduction

Heavy metal (HM) contamination of soil is a major concern due to its intrinsic toxicity to living species at high concentrations and bioaccumulation in humans and ecosystems through food chains. HM reduces the productivity of primary producers, such as plants and cultivars, by interfering with their growth and development. HMs also induce adverse physiological and morphological alterations in plants, by creating reactive oxygen species (ROS), limiting metabolic enzymes and photosynthesis, and spending energy for chelation, transport, and dislocation of absorbed HMs [1,2,3]. It is commonly understood that non-essential metals in soil, such as As, Cd, Ni, and Pb are extremely toxic to plants.

However, certain essential metals such as copper (Cu) and zinc (Zn) are toxic to plants at higher concentrations. Copper is a cofactor of several enzymes involved in photosynthesis, respiration, and ROS scavenging, and is a key micronutrient in the maintenance of cellular functions. Cu cytotoxicity causes plant damage, reduced root and shoot growth, chlorosis, and a decrease in nutrient (Fe, Mn, and nitrogen) uptake in roots when it is present in excess. Furthermore, oxidative stress caused by the production of reactive oxygen species (ROS) causes macromolecular damage [4,5,6]. At excessive quantities, Zn is also toxic to plants. Chlorosis, Fe and Mn deficiency [5], peroxidases inactivation, and root development suppression [7] are all symptoms of Zn poisoning.

In many HM contaminated sites, soils have been contaminated with multiple elements rather than a single element. Agricultural soils, different mines and mine tailings, smelters, and railroad sides are among the places [8,9,10,11,12,13]. Because of synergistic interactions, multiple contaminants exhibit more severe toxic effects on human health and the environment than a single compound. Combinations of metal and pesticide on test animals [14], metal and sodium salt on freshwater microalgae [15], and metal and metal on plants [16,17] have all shown that when multiple pollutants coexist in the environment, toxicity increases. These findings imply that for sites with numerous pollutants, it is important to consider the synergistic effects of multiple contaminants throughout the risk assessment process and to set remediation goals appropriately.

**Figure 1:**
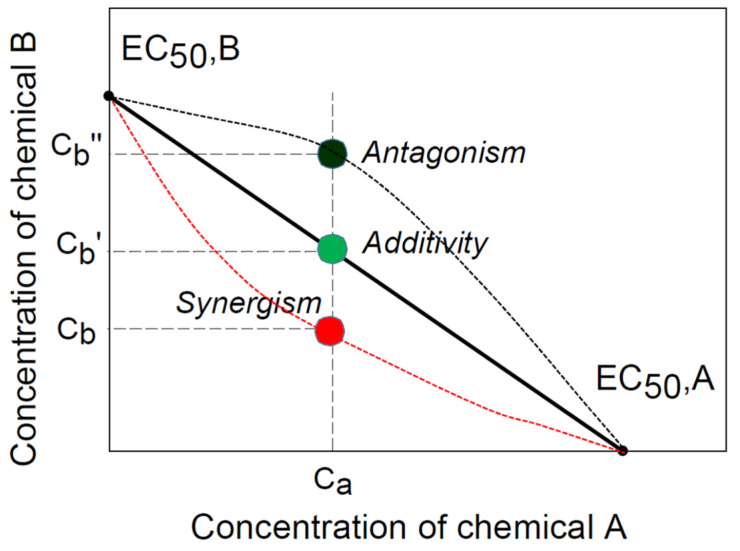
An illustration of isoboles for the analysis of interactions of two chemical compounds with synergism, additivity, and antagonism. The concentration of chemical A is fixed at a concentration of Ca in combination with different concentrations of chemical B at Cb for synergism, Cb’ for additivity, and Cb” for antagonism, all of which result in the same 50% effect.

The isobolographic method (Figure 1), proposed by Loewe in 1953, is widely used in pharmacology for the assessment of drug interactions [18,19,20,21] and may also be used to quantify the synergistic effect of two chemical compounds on plants [15]. The x- and y-axes in Figure 1 denote the concentrations of chemicals A and B, respectively. The 50% effective concentration (EC50) of any chemical constituent may be determined and plotted on the appropriate axis using dose–response relationships. By connecting two locations with a line, a 50% isobole is completed. The locus of the combination of two chemicals with a concentration of Ca + Cb’ producing the same EC50 will follow the black line passing through the green point if the interaction is additive. If the interaction is synergistic, the locus will trace the inward curve with a red point that corresponds to a lower concentration of Ca + Cb than the additivity, whereas the opposite antagonistic interaction will trace the outward curve with a black point that corresponds to a higher concentration of Ca + Cb” than the additivity.

A combination index (CI) is another method to analyze interactions. If CI > 1, the interaction is antagonistic; if CI = 1, the interaction is additive; if CI < 1, the interaction is synergistic [18].

If Cu and Zn are both toxic to *Arabidopsis thaliana* and their effects are synergistic at environmentally relevant concentrations, the degree of increased toxicity can be quantified using an isobolographic method and biometric measurements including pigment concentrations or root length. To evaluate the degree of synergistic toxic effects of two essential metals (Cu and Zn) on the plant, we used *A. thaliana* growing on an artificially contaminated metal medium in a controlled environment. In terms of total root length, the EC50 values of individual metals or combinations of metals were estimated. The value of the combination index, which determines the degree of synergistic effect, was calculated using the isobolographic method and estimated EC50 values. The quantitative value CI of this study can assist researchers to better understand the importance of synergistic metal toxicity increase to *A. thaliana*. The finding of this study can be applicable to other plants because we used a model plant (*A. thaliana)*. Furthermore, the study’s calculated CI values could be utilized to support more sophisticated estimation of metal toxicity in the ecosystem.

## 2. Materials and Methods

### 2.1. Plant Material

Seeds of *A. thaliana* (ecotype wild) were received from Prof. Ilha Lee of Seoul National University. The seeds were planted in pots containing nursery soil amended with 10% vermiculate and grown in a plant growth chamber at 25 °C with 60% humidity under long day conditions (day 16 h/night 8 h). An Apogee MQ-500 quantum meter was used to measure photosynthetically active radiation (PAR) every three days (Logan, UT, USA). The value of PAR was 145 ± 13 (*n* = 36) µmol·m^−2^·s^−1^. After 2 months of cultivation, the seeds of *A. thaliana* were harvested, screened, cleaned, and stored at 4 °C until use.

Plants on agar plates were cultivated by the method described by Remy and Duque (2016) [22] with minor modifications. In full strength Murashige and Skoog (MS) medium [23], 0.1 g/L *myo*-inositol and 0.5 g/L 2-(N-morpholino) ethanesulfonic acid (MES) were added and then the pH of the medium was adjusted to 5.7 with 1 M KOH. External Cu and Zn, individually or in combination, were added to the medium along with 0.8% plant agar (HM medium). The artificially supplied HMs are labeled as “external” since MS media includes 32.8 mM of Zn and 0.1 mM of Cu as essential metals. Each HM had a concentration of Cu and Zn of 7, and the combinations had a concentration of 9 along with a control which received no external HM. The combinations of Cu and Zn consisted of Cu 25, 50, and 75 µM, and Zn 20, 50, and 100 µM. The concentrations of the two HMs were carefully chosen after comparison with prior investigations [16,17,22]. After sterilization of the MS medium via autoclave, 4 mg/L of filter sterilized fenbendazole and streptomycin were added to make agar gel in a square Petri dish (120 mm × 120 mm × 20 mm).

Seeds were surface sterilized with 50% (*v/v*) NaOCl and 0.02% (*v/v*) Triton X-100, then subsequently rinsed five times with sterile deionized water (18.3 MΩ-cm). To break dormancy, the sterilized seeds were placed on agar medium that had not been spiked with external HM (control), sealed with breathable Micropore tape (3M, St. Paul, MN, USA), and kept at 4 °C in the dark for three days. After 3 days, the Petri dish was placed vertically in a growth chamber under the same growth conditions described above until the root length of *A. thaliana* reached approximately 1 cm. The germinated seedlings were carefully transplanted to the control and HM media (5 seedlings per each) in triplicate and cultivated for 8 days in a growth chamber. The same experiment was repeated twice within 6 months. For each treatment, a total of 30 seedlings were tested.

### 2.2. Measurement and Analysis

At the end of the experiment, the Petri dish was photographed with a high-resolution camera (Canon G7X Mark II, Tokyo, Japan), and the above-ground part was harvested, weighed, and extracted with 5.0 mL of 80 percent acetone to measure chlorophyll-a (Chl-a), chlorophyll-b (Chl-b), and carotenoid concentrations [24]. ImageJ software was used to convert the photos to an 8-bit RGB format, which was then used by SmartRoot software to assess the total root length (TRL) [25,26].

### 2.3. Statistical Analysis

The measured values of pigment concentration and TRL showed a lot of variation. The extreme studentized deviation (ESD) method [27] in GraphPad software was used to reduce outliers [27]. Because a plant’s measured Chl-a, Chl-b, carotenoid, and TRL values were considered a set, if one of the sets’ values was found to be an outlier, the entire set of data was discarded. After data reduction, the four-parameter logistic equation, as shown in Equation (1), was used to estimate EC50. Using the Quest Graph^TM^ IC calculator, the TRL data of individual or combinations of HMs was fitted to Equation (1) to estimate the EC50 [28].

(1) $$y=d+\frac{a-d}{\left[1+{\left(\frac{x}{c}\right)}^{b}\right]}$$

where *a* = theoretical response at zero concentration; *b* = slope factor; *c* = mid-range concentration; *d* = theoretical response at maximum concentration.

## 3. Results and Discussion

### 3.1. Effects of Heavy Metals on Seedling Growth

**Figure 2:**
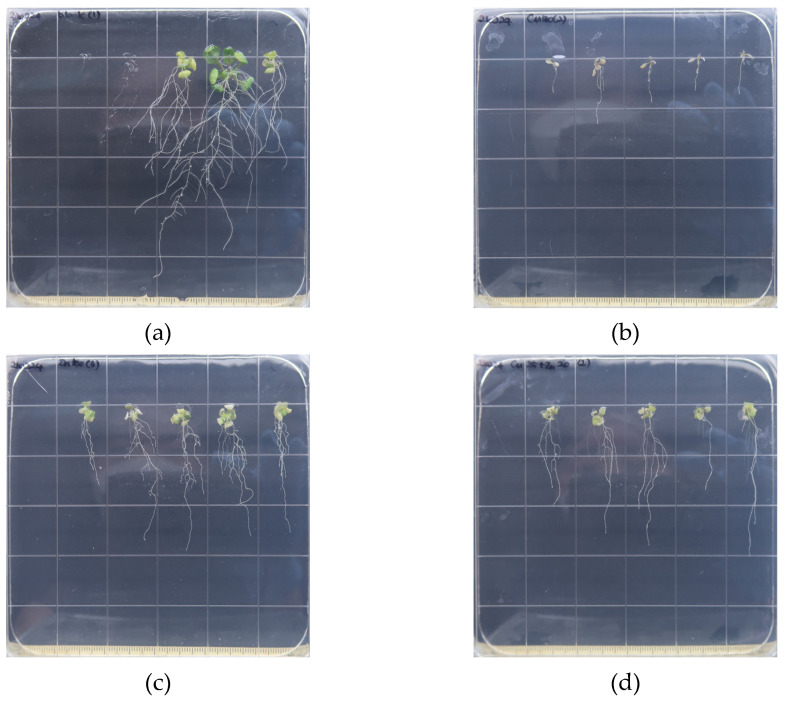
High-resolution images of *A. thaliana* seedling growth after 8 days on the agar medium in a controlled environment with varied metal treatments. The dimension of each square in the Petri dish is 2 cm × 2 cm. (**a**) The control medium without external metal; (**b**) external Cu 100 μM; (**c**) External Zn 100 μM; (**d**) combination of external Cu 25 μM + Zn 20 μM.

After 8 days on the agar medium with varied metal treatments, high-resolution images of *A. thaliana* seedling growth were taken (Figure 2). A high concentration (100 mg/L) of streptomycin inhibited the growth of *A. thaliana* plants in an agar medium by destructing chlorophyll [29]. However, no indications of chlorosis were found in the control medium amended with 4 mg/L streptomycin. In contrast to the HM media, primary and secondary roots, as well as leaves, developed quickly. As shown in Figure 2a, some seedlings grew irregularly faster on the control medium, necessitating outlier elimination.

Growth and root elongation were greatly hindered under the Cu 100 μM treatment with the development of chlorosis in leaves as shown in Figure 2b. Zn did not inhibit primary and lateral root development as much as Cu, but it stunted it slightly. In the Cu 25 μM + Zn 20 μM combination treatment (Figure 2d), inhibition of leaf growth and lateral root development was more severe than that of Zn 100 μM, despite the total concentration of HMs being less than 100 μM. In other studies, the average root length of *A. thaliana* treated with Cu 50 μM was comparable to that treated with Zn 200 μM [16]. The concentration of 25% of seedling growth inhibition occurred at approximately 20 μM of Cu while Zn was at 500 μM [7]. These results suggest that the inhibition of Cu on seedling growth is at least 4 to 25 times higher than that of Zn.

**Figure 3:**
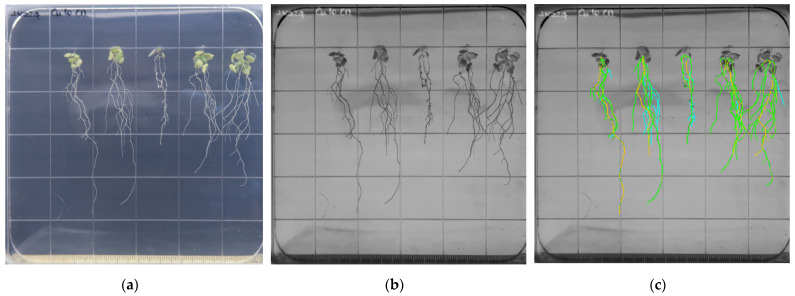
Image processing procedures for the estimation of the total root length of *A. thaliana* cultured in a growth chamber for 8 days on an agar medium: (**a**) original high resolution; (**b**) image conversion to 8-bit RGB format by ImageJ; (**c**) SmartRoot semi-automatic root length estimation. The primary root (yellow), secondary (green), and tertiary (blue) roots are all color-coded.

There are three steps in the TRL estimation. When taking a high-resolution photograph at a fixed length, a forensic scale was placed alongside the agar gel (Figure 3a). The photo was converted to an 8-bit RGB format using ImageJ (Figure 3b). Before root estimation, the SmartRoot program set a DPI value of 1 cm on a scale, and the primary root was traced by placing a dot on the contrasted root image, followed by secondary and tertiary roots (Figure 3c).

### 3.2. Variations in Pigment Concentration and TRL

The measured values of pigment concentration and TRL are summarized in Table 1 along with external HM concentrations. The number of plants varies because of plant senescence after seedling transplant and outlier elimination. Overall, the values decreased as the HM concentration increased, either individually or in combination of Cu and Zn. Complete pigment concentration and TRL data is presented in Table S1 (please refer Supplementary Materials).

The above-ground pigment concentrations varied far more than the TRL concentrations, as demonstrated by the CV values. The CV of TRL is 41.2 percent, whereas the CVs of Chl-a, Chl-b, and carotenoid are 78.0, 69.0, and 71.7 percent, respectively. When the concentration of carotenoids was gradually increased from 100 μM to 400 μM, the concentration of carotenoids decreased. A similar varied carotenoid response was observed in Cu supplemented *A. thaliana* [16]. Despite the fact that shoot growth and pigment concentration are useful indicators of plant metal sensitivity [22], more exact methodological research may be required to decrease variability.

**Figure 4:**
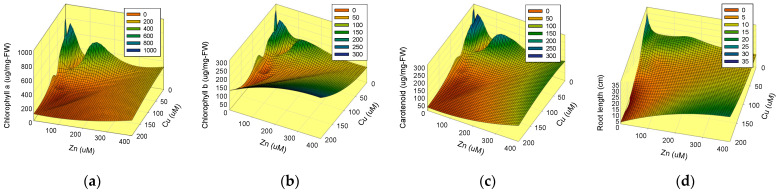
The changes in the aboveground pigment concentration and total root length of *A. thaliana* after 8 days of growth on agar media containing individual and combinations of HMs (Cu and Zn) in a plant growth chamber at vertical position. (**a**) Chlorophyll a; (**b**) chlorophyll b; (**c**) carotenoid; (**d**) total root length.

The effects of HMs on pigment concentration and TRL are depicted in a three-dimensional graph (Figure 4). In all pigment graphs, there is a concave zone in the lower concentration of Cu 50 μM + Zn 20 μM. Copper demonstrated more toxicity, as evidenced by a drop in pigment content, than Zn; however, Chl-b concentration rose from 20 μM to 50 μM under the Zn treatment. The inconsistency may have arisen from an error made when cutting the above-ground section of the plant, which is difficult to distinguish from the root.

The TRL in Figure 4d showed more consistent and continuous results than pigment concentrations. It decreased along the x-and y-axes, and a concave zone appeared in the central region, indicating that two HMs have synergistic toxic effects on TRL. Previous studies found that at 300 μM, *A. thaliana* shoot and root growth were significantly slowed, and chlorosis was caused by Fe absorption inhibition caused by excess Zn [30,31]. In hydroponic culture, root development of *A. thaliana* was reduced starting at Cu 25 μM and entirely stopped at Cu 50 μM due to root meristem damage [32]. The main and lateral root length development were the most effective indicators of HM toxicity to *A. thaliana* grown on a Petri dish [33]. For these reasons, EC50 is estimated based on TRL.

Cu toxicity in plants is caused by interfering with various physiological plant growth processes, such as germination, root and shoot growth, damage to the photosynthetic apparatus, and an increase in reactive oxygen species (ROS), which increases protective mechanisms of enzymatic and non-enzymatic antioxidant activity [33]. Among these toxic effects, Cu critically damages root development. Plants of *Hymenaea courbaril* exposed to excess Cu (800 mg/kg-soil) showed a reduction in length, surface area volume, and dry mass of roots [34]. *A. thaliana* grown in either a gel medium or hydroponics on an excess Cu concentration showed changes in lignin accumulation and root length reduction, or significant changes in root morphology [32,35,36]. Another study with Rhodes grass in resin buffered hydroponics showed that less than 1 μM of external Cu resulted in a reduction in root growth [37]. Excess Zn also harms plants by causing stunted growth, decreased photosynthesis and respiration, nutrient deficiency, and ROS production. Among those toxic effects, reduction in root growth, especially radicle elongation, was mainly caused by disruption of the nucleus in root tip cells [38,39]. These effects may have been attributed to inhibition of peroxidase by Zn and a resulting failure in the sequestration of ROS [7]. Zn also interfered with auxin accumulation in the root thus inhibiting the growth of the primary root of *A. thaliana* [40]. Overall, previous research indicates that total root length may be an important indicator of HM toxicity in plants.

### 3.3. Estimation of EC 50 and Combination Index

The 4-parameter logistic equation was used to predict Cu and Zn EC50 values based on TRL, as shown in Table 2. Cu and Zn had EC50 values of 40.0 and 76.4 μM, respectively. The results of data fitting to the logistic equation is presented in Figure S1 (please refer Supplementary Materials). The EC50 results clearly demonstrated that *A. Thaliana*’s growth was hindered more on the Cu medium than on the Zn medium. The plant was administered to Cu at concentrations ranging from 0 to 100 μM in a hydroponic culture, and the EC50 value for root elongation was found to be around 10 μM [36]. However, the EC50 values for Cu and Zn in terms of primary root reduction were approximately 50 μM and 250 μM, respectively, in another meta-analysis [41]. The values varied depending on the culture conditions and analysis methods, but reductions in primary root length and changes in lateral root density are common [42,43]. Therefore, more study efforts are necessary to accurately determine the EC50 values of HM for the plant.

**Figure 5:**
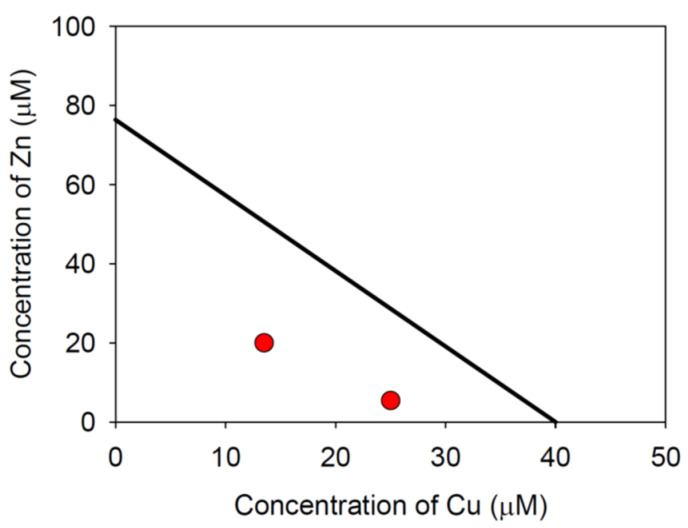
The estimated EC50 isobole of Cu and Zn in comparison to two red dots showing synergistic toxic effects. The straight line of isobole is constructed using the EC50 values of Zn and Cu estimated from the results of single heavy metal experiments. Equation (4) was used to obtain two red dots from the results of a combination of two HM experiments.

Based on EC50, an isobologram (Figure 5) is constructed by Equation (1):

(2) $$\frac{Cu}{EC50,\;Cu}+\frac{Zn}{EC50,Zn}=1$$

The non-constant combination ratio approach [20] was used to determine the values of EC50 for the combined Cu + Zn. With a constant Cu (25 μM) or Zn (20 μM) concentration, the Zn or Cu concentrations were changed (Table 1), and the EC50 on TRL was calculated. Cu 20 μM with a varied Zn concentration from 20 to 100 μM had an EC50_Cm2_ of 25.4 μM; while Zn 25 μM with a varied Cu from 25 to 75 μM had an EC50_Cm1_ of 33.5 μM. These figures are significantly lower than those obtained with a single HM, implying strong synergistic toxicity when Cu and Zinc are combined.

The equation of the combination index (CI), a numerical value defining the interaction, is in Equation (3):

(3) $$\mathrm{CI}=\frac{D_{Zn}}{EC50,\;Zn}+\frac{D_{Cu}}{EC50,Cu}$$

where *D* denotes the concentration of each HM in combination.

From the combined EC50 values, CI was calculated by Equation (4) and summarized in Table 3:

(4) $$\mathrm{CI}=\frac{D_{Zn}}{EC50,\;Zn}+\frac{EC50_{Cm}-D_{Zn}}{EC50,Cu}$$

where *D_Zn_* = concentration of fixed concentration of Zn; *C_m_* = the total of Cu and Zn concentrations in combination.

Both cases had CI values smaller than 1.0, indicating that Zn and Cu have a synergistic effect on the root growth of *A. thaliana*. The corresponding points are presented in red dots (Figure 5). Other tested concentrations higher than Zn 25 μM and Cu 20 μM were above the isobole in Figure 5. Because the fixed-ratio two HM was not employed in the trials [37], the overall combination index [36] could not be determined in this study. The findings of this investigation, however, clearly shown that Zn and Cu had synergistic toxicity on growth of *A. thaliana*.

Many studies have found that the combination of Cu and Zn is more toxic to plants. When three Brassica species were exposed to Cu (0.35 mg/L) and Zn (6.5 mg/L) in hydroponic reactors, the reduction in shoot and root biomass was twice as great as when Zn was used alone [44]. The same observations were reported in *A. thaliana* grown in soil [16], and in a maize plant spiked with 90 mg/kg of Zn in Cu contaminated vineyard soil [45]. However, quantitative values describing the interactions have not been reported. Unlike the previous study, the synergistic effects were estimated and a numerical value was provided in this study.

## 4. Conclusions

To evaluate the interaction of heavy metals, we exposed a model plant, *Arabidopsis thaliana,* to individual or combinations of Cu and Zn in agar medium and cultured it in a controlled growth chamber for 8 days. Total root length and pigment concentrations in the above-ground section, including chlorophyll-a, chlorophyll-b, and carotenoid, were measured. The decrease in pigment concentration was not consistent with the increase in metal concentration. Furthermore, the coefficients of variation in the measured values of pigments were twice that of the total root length. For these reasons, total root length was used to predict heavy metal toxicity in *A. thaliana*, and EC50 values were calculated using total root length as an indicator.

The estimated EC50 for Cu and Zn from individual metal exposure was 40.0 and 76.4 μM, respectively. When the plant was exposed to metals in combinations, the estimated EC50 for 25 μM Zn with varied Cu was 33.5 μM and the EC50 for 20 μM Cu with varied Zn was 25.4 μM. The combination index for the 20 μM Cu and 25 μM Zn was 0.63 and 0.60, respectively. These combination indexes of less than 1.0 with the metals in combination proved that the combination of metals exerts synergistic toxicity on the plant.

The study showed that the isolographic method can be a useful and effective tool for the quantitative estimation of the synergistic toxicity of two heavy metals on plants. However, more rigorous and better experimental studies, such as the fixed-ratio isobologram method, may provide a better understanding of the synergistic interactions.

## Figures and Tables

**Table 1:** The average concentration of pigments in the aboveground part and the total root length of *A. thaliana* grown for 8 days on the respective MS medium containing varied concentrations of individual or combinations of Cu and Zn. The values are the mean ± standard deviation.

Cu (μM)	Zn (μM)	N ^†^	Chl-a (μg/g-F.W.)	Chl-b (μg/g-F.W.)	Carotenoid (μg/g-F.W.)	Total Root Length (cm)
0	0	19	795.0 ± 411.2	263.4 ± 111.3	269.7 ± 127.5	30.1 ± 11.8
5	0	19	727.0 ± 393.2	232.0 ± 111.4	248.1 ± 124.1	25.3 ± 10.3
10	0	30	398.1 ± 364.9	122.7 ± 110.8	146.1 ± 115.6	23.9 ± 8.1
25	0	23	361.0 ± 379.4	115.1 ± 112.5	129.1 ± 120.9	20.4 ± 6.6
50	0	21	248.1 ± 191.4	85.2 ± 51.8	101.8 ± 70.7	11.5 ± 5.0
75	0	20	235.7 ± 173.1	118.0 ± 118.9	94.5 ± 73.0	6.2 ± 2.2
100	0	19	174.8 ± 106.6	75.4 ± 63.1	66.0 ± 38.0	3.1 ± 1.2
200	0	21	98.3 ± 43.5	117.5 ± 98.3	22.6 ± 13.4	1.5 ± 0.5
0	10	20	360.5 ± 299.9	118.0 ± 72.0	134.6 ± 102.3	19.9 ± 7.6
0	20	19	489.7 ± 316.5	150.9 ± 75.3	176.5 ± 109.7	16.3 ± 5.2
0	50	19	481.7 ± 423.6	167.4 ± 120.9	169.2 ± 146.7	14.0 ± 5.0
0	100	20	321.5 ± 298.8	131.8 ± 63.4	113.3 ± 102.7	14.4 ± 3.4
0	150	22	419.0 ±304.7	130.4 ± 66.9	154.1 ± 103.6	16.1 ± 3.2
0	200	20	364.4 ± 286.3	115.9 ± 67.6	132.5 ± 96.5	14.3 ± 2.2
0	400	20	316.7 ± 200.4	92.5 ± 52.5	128.2 ± 66.2	10.3 ± 3.7
25	20	22	460.5 ± 377.3	146.0 ± 103.0	160.4 ± 119.5	13.2 ± 5.8
25	50	30	334.5 ± 352.7	109.2 ± 89.5	126.0 ± 125.3	15.1 ± 6.5
25	100	26	427.7 ± 367.9	134.2 ± 95.9	152.1 ± 116.1	14.4 ± 6.2
50	20	27	237.7 ± 209.4	77.9 ± 57.6	98.7 ± 76.2	11.5 ± 5.6
50	50	22	301.6 ± 290.3	100.9 ± 80.3	119.4 ± 103.6	11.8 ± 4.9
50	100	20	254.7 ± 238.4	86.4 ± 63.3	102.8 ± 86.4	8.6 ± 3.2
75	20	21	99.8 ± 73.7	34.6 ± 22.7	45.5 ± 21.5	9.0 ± 6.8
75	50	21	117.5 ± 63.2	48.9 ± 26.6	50.8 ± 23.4	7.7 ± 6.3
75	100	24	140.0 ±128.3	66.1 ± 52.9	58.4 ± 52.0	7.0 ± 5.3
CV ^‡^	-	-	78.0	69.0	71.7	41.2

^†^ the number of plants, ^‡^ coefficient of variation.

**Table 2:** Estimated EC50 values in terms of total root length of *A. thaliana* after 8 days of growth on an agar media containing individual and combined Cu and Zn. The EC50 is based on a 4-parameter logistic model.

EC50_Cu_	EC50_Zn_	^1^ EC50_Cm1_	^2^ EC50_Cm2_
40.0 μM	76.4 μM	33.5 μM	25.4 μM

^1^ Zn 25 μM+ Cu (0, 25, 50, 75 μM), ^2^ Cu 20 μM+ Zn (0, 20, 50, 100 μM).

**Table 3:** Result of combination index calculation. Based on the results of the Cu + Zn combination EC50, values for the combination index were calculated using Equation (4).

^1^ CI_Cm1_	^2^ CI_Cm2_
0.60	0.63

^1^ Zn 25 μM+ Cu (0, 25, 50, 75 μM), ^2^ Cu 20 μM+ Zn (0, 20, 50, 100 μM).

## Data Availability

The full data of the study are available on request to the corresponding author.

## Supplementary Materials

The following supporting information can be downloaded at: https://www.mdpi.com/article/10.3390/toxics10040195/s1, Figure S1: EC50 estimated by the 4-parameter logistic equation; Table S1: Concentration of pigments in the above-ground part and total root length values from the SmartRoot program estimation.
